# Characteristic Facial Expressions During Visual Fixation in Japanese Older Adults With Lower Cognitive Screening Performance: Exploratory Cross-Sectional Study

**DOI:** 10.2196/99948

**Published:** 2026-10-27

**Authors:** Mirano Miyayama, Takuya Sekiguchi, Hikaru Sugimoto, Toshikazu Kawagoe, Kornkanok Tripanpitak, Alexandra Wolf, Kazumi Kumagai, Kosuke Fukumori, Kumi Watanabe Miura, Shogo Okada, Keiichiro Ishimaru, Mihoko Otake-Matsuura

**Affiliations:** 1 Department of Developmental and Clinical Psychology, The Division of Human Developmental Sciences, Graduate School of Humanities and Sciences, Ochanomizu University, Tokyo, Japan; 2 RIKEN Center for Advanced Intelligence Project, Nihonbashi 1-chome Mitsui Building, 15th floor, 1-4-1 Nihonbashi, Chuo-ku, Tokyo, 103-0027, Japan, 81 3-6225-2482; 3 Japan Society for the Promotion of Science, Tokyo, Japan; 4 Centre for Human Brain Health, School of Psychology, University of Birmingham, Birmingham, United Kingdom; 5 Institute for Mental Health, School of Psychology, University of Birmingham, Birmingham, United Kingdom; 6 School of Humanities and Science, Kumamoto Campus, Tokai University, Kumamoto, Japan; 7 Department of Neuropsychiatry, Graduate School of Medical Sciences, Kyushu University, Fukuoka, Japan; 8 Japan Advanced Institute of Science and Technology, Ishikawa, Japan; 9 Faculty of Core Research, Ochanomizu University, Tokyo, Japan

**Keywords:** cognitive decline, cognitive screening, facial action units, OpenFace, facial analysis, digital biomarker, early detection, older adults

## Abstract

**Background:**

Early identification of older adults with lower cognitive screening performance may facilitate appropriate clinical evaluation and intervention to delay the progress of cognitive decline and onset of dementia. Because facial information can be acquired noninvasively and unobtrusively during everyday life, it has attracted increasing attention as a potential digital biomarker. However, it remains unclear which facial regions are associated with cognitive decline under a neutral facial condition. Furthermore, it is unknown whether these facial characteristics primarily reflect static facial appearance or facial movements.

**Objective:**

This study aimed to identify facial action unit (AU) patterns associated with lower cognitive screening performance during a fixation-cross viewing condition that is less susceptible to the influence of language and emotional elicitation.

**Methods:**

Older adults were classified into either a lower cognitive screening performance group (Montreal Cognitive Assessment Japanese version [MoCA-J] score ≤25; n=31) or a healthy control group (MoCA-J score ≥26; n=14). Facial videos were recorded while participants viewed a fixation cross for 1 minute. OpenFace 2.0 was used to extract the intensities of 17 AUs and the presence of 18 AUs. Video analyses were first performed to identify AU characteristics associated with cognitive screening performance. For AU intensity, frame-level mixed-effects models with participant-specific random intercepts and a first-order autoregressive (AR(1)) correlation structure were used, adjusting for age, Geriatric Depression Scale score, and sex, and participant-level sensitivity analyses were conducted to assess the consistency of the frame-level findings. For AU presence, participant-level analyses were performed because the frame-level models did not yield reliable parameter estimates. Subsequently, single-frame analyses were performed to examine whether the AU characteristics identified in the video analyses could be explained solely by static facial appearance. As this study was exploratory and hypothesis-generating, *P* values were not adjusted for multiple comparisons.

**Results:**

For AU intensity, frame-level video analyses showed that the lower cognitive screening performance group had a higher probability of zero intensity for AU10 (upper lip raiser), with participant-level analysis based on the proportion of zero-intensity frames yielding consistent results. In contrast, single-frame analyses of AU intensity showed no significant group difference for AU10, suggesting that the observed difference may reflect facial movements rather than static facial appearance. For AU presence, video analyses showed no significant group differences.

**Conclusions:**

Older adults with lower cognitive screening performance exhibited distinct AU patterns related to AU10 (upper lip raiser) during fixation-cross viewing, which may reflect subtle differences in facial movements around the mouth. These exploratory findings identify candidate facial biomarkers associated with lower cognitive screening performance and warrant validation in larger, balanced, independent samples.

## Introduction

Mild cognitive impairment (MCI) is a prodromal stage of dementia characterized by cognitive decline that cannot be attributed to normal aging. However, it does not meet the diagnostic criteria for dementia, and individuals with MCI typically maintain their activities of daily living [1]. Despite preserved daily functioning, the extent of brain lesions in this population is intermediate between that of healthy individuals and patients with dementia [2].

Some people with MCI progress to dementia, with annual rates of around 10% [3]. As populations around the world continue to age, the prevalence of MCI is becoming an increasingly critical public health issue. In Japan alone, the number of individuals with MCI is estimated to be around 5.58 million [4], underscoring the urgency of addressing this challenge.

Despite these observations, a proportion of individuals with MCI may revert to normal cognitive function [5]. For instance, Chung et al [6] reported that 39% of individuals with MCI returned to a cognitively healthy state. In the absence of curative treatments for dementia, early detection and timely intervention at the MCI stage remain essential [7,8].

The early detection of MCI remains challenging [7]. One contributing factor is that current methods for detecting MCI are not easily accessible, as they often require specialized neuropsychological assessments, neuroimaging, and evaluation in clinical settings [9], which may impose a burden on patients. Because cognitive performance decline often does not interfere with activities of daily living in its early stages, individuals may remain unaware of these changes [10]. These limitations highlight the need for more accessible approaches that can help identify older adults who may benefit from formal cognitive evaluation in everyday settings. Such approaches are intended not to replace the clinical diagnosis of MCI or established cognitive screening tests, but rather to encourage older adults with possible but unrecognized cognitive decline to undergo comprehensive cognitive assessment. By enabling more natural and unobtrusive detection in everyday settings, they may provide an opportunity for older adults who might otherwise remain unaware of their cognitive decline to seek appropriate clinical evaluation.

One promising approach is the use of facial information, which can be acquired noninvasively and unobtrusively in everyday environments. Rather than replacing clinical diagnosis or established cognitive screening tests, facial information may provide candidate facial biomarkers associated with cognitive decline. The identification of such biomarkers may provide a scientific foundation for future digital prescreening approaches capable of identifying older adults who could benefit from subsequent cognitive assessment. Facial expressions are considered important information in clinical psychiatry as an adjunct to diagnosis [11]. Objectively quantifying facial features used to be difficult, but recent technological advancements have made this more possible, and characteristic facial expressions specific to mental disorders have been the focus of several recent studies [12,13]. For example, Jung et al [14] found that characteristic facial expressions, such as lip press and stretch, were attenuated in patients with depression compared to those in healthy controls (HCs).

Characteristic facial expressions have been reported not only in mental disorders but also in dementia. For example, in patients with Alzheimer disease (AD), when they view a sequence of emotion-eliciting and neutral images, it has been noted that their degree of cognitive decline corresponds to increased facial expressiveness [15]. Additionally, Burton and Kaszniak [16] reported that while viewing emotion-eliciting images, zygomatic activity was different between AD and control groups. It has also been reported that AI, deep learning, or machine learning approaches can distinguish between dementia and healthy groups based on video-recordings of facial expressions during conversations and when participants are passively viewing images [17-20].

Characteristic facial expressions associated with dementia have also been reported using AI-based image analysis. In particular, Umeda-Kameyama et al [21] demonstrated that a deep learning model using a single frontal photograph of a neutral face could discriminate individuals with dementia from HCs with high accuracy. Furthermore, the Face AI score generated by the model was more strongly associated with cognitive impairment than with age, suggesting that the AI captured facial characteristics related to cognitive impairment rather than merely age-related facial changes.

Taken together, these findings raise the possibility that facial characteristics associated with cognitive decline may also be present in individuals in the early stages of cognitive decline, even when they are exhibiting neutral facial expressions. However, because the deep learning model of Umeda-Kameyama et al [21] is inherently a black box, it remains unclear which facial features contributed to the classification. The authors speculated that the model may have relied not on facial movements themselves but on appearance-related characteristics, such as wrinkles and features around the mouth. Nevertheless, the specific facial features underlying the classification have not yet been identified.

One promising and more interpretable approach to addressing this issue is the use of action units (AUs). AUs are not direct measures of facial muscle activity; rather, they represent estimated facial actions inferred from changes in facial appearance that are conventionally associated with the activation of individual facial muscles or muscle groups [22]. Compared with black-box AI models, AU-based analyses provide greater interpretability by enabling researchers to identify which facial characteristics contribute to group differences.

OpenFace [23] is a widely used open-source facial behavior analysis toolkit that uses machine learning models to estimate and quantify AUs from facial images and videos. It has been used in previous studies investigating facial characteristics associated with dementia and cognitive impairment (eg, [17,19]). OpenFace has also been applied to video-based facial analysis in older adults, including patients with AD and vascular dementia [24]. Using OpenFace, Ko et al [25] analyzed 15-second videos of Korean older adults maintaining a neutral facial expression and reported that multiple AUs were present even under neutral facial conditions. They also discussed the possibility that the AUs identified in their study could reflect either stable facial characteristics or subtle facial movements. However, because OpenFace estimates AUs independently for each frame using facial appearance and landmark-based facial shape features, rather than explicitly modeling facial motion, the study could not determine whether the features identified through video analysis reflected a stable facial appearance and morphology, transient facial movements, or a combination of these.

Most previous studies investigating facial characteristics associated with cognitive impairment have evaluated facial expressions during conversations or emotion-eliciting tasks ([15-17,19,20]). Although these paradigms provide valuable information, facial behavior during such tasks may be influenced not only by cognitive impairment itself but also by social interaction, emotional responses, and the participant’s ability to understand and perform the task. In contrast, facial characteristics during fixation-cross viewing can be evaluated under a standardized, low-demand condition that minimizes these influences while requiring only minimal effort. Such an approach may also provide a practical framework for identifying candidate facial biomarkers that may ultimately contribute to future facial-information–based digital prescreening approaches.

To our knowledge, no previous study has investigated specific AU characteristics associated with early cognitive impairment during a non-emotional fixation condition.

Therefore, we investigated whether older adults classified as having lower cognitive screening performance exhibit characteristic AU patterns while viewing a fixation cross. We first analyzed AU patterns across facial videos to identify facial characteristics associated with lower cognitive screening performance. Then, we performed an exploratory single-frame analysis to determine whether the identified AU patterns reflected facial characteristics already present in the participant’s initial facial appearance or emerged from facial movements during the recording. Rather than developing or validating a facial information-based digital screening tool, our work was exploratory and aimed at identifying candidate facial biomarkers associated with lower cognitive screening performance. This is conceived of as a first step toward the biomarkers’ subsequent validation and the future development of facial-information–based digital prescreening approaches.

## Methods

### Study Design

A cross-sectional study design was adopted because the aim of this study was to identify facial features associated with cognitive screening performance at a single time point, not to examine facial features associated with longitudinal changes in cognitive screening performance or those predictive of future screening performance. In addition, because facial AU characteristics during fixation-cross viewing have not been investigated in relation to cognitive screening performance, we adopted an exploratory approach to identify candidate facial features across multiple AUs rather than test hypotheses regarding specific AUs.

### Participants

A total of 50 participants (19 male and 31 female) were recruited for the study. Inclusion criteria were (1) age 65 years or older and (2) being a native Japanese speaker. Exclusion criteria included (1) use of medications that affect cognitive function, (2) a history of neurological or psychiatric disorders that may impact the central nervous system, and (3) a history of severe head trauma.

### Study Setting and Recruitment

Participants were recruited through 3 sources: individuals who had previously participated in research conducted by our research group, a participant recruitment company, and a Silver Human Resources Center, a Japanese organization that provides work opportunities to registered older adult members. Recruitment flyers were mailed to previous research participants and were also used for recruitment through the participant recruitment company and the Silver Human Resources Center. Individuals who expressed interest in participating were provided with information about the study, and those who provided written informed consent participated in the study.

The study was conducted in a research room at the RIKEN Center for Advanced Intelligence Project and in rented meeting rooms in Tokyo, Japan. Magnetic resonance imaging (MRI) data were collected at the Advanced Imaging Center Yaesu Clinic, Tokyo. Data were collected between December 2024 and March 2025.

The target sample size was set at 50 participants based on resource constraints, including the available study period and budget for data collection. No a priori power analysis was conducted because no previous studies had examined the association between facial AU characteristics during fixation-cross viewing and cognitive function, and therefore there was insufficient prior evidence to specify an expected effect size for such an analysis. Accordingly, the findings of this exploratory study should be considered preliminary.

### Procedure

To comprehensively characterize cognitive decline across various domains, a multimodal dataset was acquired, including facial data captured during fixation cross viewing, conversation, and a facial expression task in which participants were instructed to voluntarily produce happy and angry facial expressions, conversational data, cognitive assessments, questionnaires, eye-tracking metrics, electroencephalography (EEG), and MRI. Data collection was conducted over a total of 3 days.

On the first day, participants completed the following procedures at the study site. First, they completed a series of facial tasks. They were initially presented with a fixation cross while their facial expressions were recorded. This was followed by the facial expression task and the conversation task. Subsequently, participants completed paper-based questionnaires containing the Geriatric Depression Scale (GDS) [26] and basic demographic items. After completing the questionnaires, cognitive assessments, including the Japanese version of the Montreal Cognitive Assessment (MoCA-J) [27], were administered face-to-face. Participants then completed EEG-based experimental tasks on the same day. MRI scanning and eye-tracking experimental tasks were conducted on separate days.

In the present study, facial data recorded while participants viewed a fixation cross, together with MoCA-J score, GDS scores, and demographic data, were used to investigate facial features associated with cognitive screening performance in a nontask condition. Analyses of the remaining modalities will be reported separately to provide further insight into the multidimensional characteristics of cognitive decline.

### Cognitive Assessment

The MoCA-J, a screening instrument for MCI with confirmed validity and reliability [27], was used. The MoCA-J is known to have higher accuracy in detecting MCI [27] and comprises 12 tasks assessing multiple cognitive domains including memory, language, executive function, visuospatial skills, calculation, abstraction, attention, concentration, and orientation [28]. Response formats vary across tasks and include verbal and performance-based components. The MoCA-J takes approximately 10 minutes to administer. Each task was scored according to the MoCA-J scoring manual, and the task scores were aggregated to obtain a total score ranging from 0 to 30 points. The recommended education correction was applied in accordance with the scoring manual. The MoCA-J was administered by one of 2 qualified psychologists, both of whom were Certified Public Psychologists and Certified Clinical Psychologists in Japan. Scoring was performed by the administering psychologist and subsequently checked by the other psychologist. Individuals with a score of 26 or above are classified as HCs, whereas those with a score of 25 or lower are classified as having suspected MCI [27]. This study followed this criterion; participants scoring 25 or below were classified as the lower cognitive performance group, whereas those scoring 26 or above were classified as the HC group. Throughout this article, participants scoring 25 or below are referred to as the lower cognitive performance group because group classification was based on cognitive screening performance rather than a clinical diagnosis of MCI.

### Assessment of Facial Expression

Participants were told, “With your eyes open, look at the fixation cross for 1 minute, and keep your mind as clear as possible.” Their facial expressions were recorded using a web camera. To minimize noise from responses to instructions and nodding, we excluded the first and last 5 seconds of the 1-minute recording and analyzed the remaining 50 seconds of facial expressions.

Facial Action Coding System (FACS) is a comprehensive, anatomically based system for describing all visually discernible facial movements [22]. It describes facial expressions by decomposing them into AUs.

OpenFace 2.0 is an open-source tool that uses machine learning models to recognize a subset of 17 or 18 AUs, specifically AU1, 2, 4, 5, 6, 7, 9, 10, 12, 14, 15, 17, 20, 23, 25, 26, 28, and 45 from videos [23,29,30]; the descriptive labels for each AU are provided in the *Results* section. OpenFace 2.0 consists of 4 major processing pipelines: facial landmark detection and tracking, head pose estimation, eye gaze estimation, and facial AU recognition. Facial landmarks are first detected and tracked, after which head pose is estimated before facial AU recognition [23].

OpenFace 2.0 quantifies the movement of each detected AU in terms of intensity and presence. AU intensity values represent the strength of each of the 17 AUs on a continuous scale ranging from 0 to 5, while AU presence values represent whether an AU is visible on the face, with 0 denoting its absence and 1 indicating its presence. The presence values are calculated for 18 AUs, consisting of the AUs used for intensity values plus AU28 (lip suck). OpenFace’s performance has been validated in previous studies [23,31]. Since the AU intensity and presence predictors were trained separately on different datasets, and their predictions may not always be consistent [29], this study used both sets of features (AU intensity and presence) and examined the consistency of the results.

The face videos recorded in this study were captured indoors in the conference room where the experiment took place using a fixed webcam (Logitech Brio 300) under stable artificial lighting conditions, with a resolution of 1920 × 1080 pixels. Participants were seated facing the camera and instructed to look at a fixation cross throughout the recording. The recording setup was designed to position participants at a distance that allowed the entire face to remain clearly visible within the camera field of view and to provide image quality suitable for facial landmark detection and AU estimation using OpenFace. As a result, each video consisted of 1250 frames, with each frame containing 17 AU intensity values and 18 AU presence values.

### GDS

Because depressive symptoms may be related to facial expressions [14] and depressive symptoms and MCI occasionally coexist [32], to prevent confounding, we assessed participants for depressive symptoms by using the Japanese version of the GDS. Its validity and reliability have also been confirmed [26]. The GDS consists of 15 questions, including “Do you feel that your life is empty?” and other questions, which are answered by choosing “yes” or “no.” Each item was scored as 0 or 1 according to the standard GDS-15-J scoring procedure, and the item scores were summed to obtain a total score ranging from 0 to 15, with higher scores indicating greater depressive tendencies. Scoring was performed by 1 author and subsequently checked by another author.

### Ethical Considerations

This study was approved by the RIKEN Ethics Committee (Wako 2022-15(2)). All participants provided written informed consent prior to participating. Facial video data were collected and treated as potentially identifiable information; therefore, they were securely stored with restricted access. The raw facial videos were used only for feature extraction using OpenFace and were not directly used in the statistical analyses. Instead, facial expression features (AU intensity and AU presence) extracted from the videos were used for all statistical analyses. These derived data were anonymized prior to analysis, and no personally identifiable information was included in the analytical dataset. In addition, questionnaire data and cognitive assessment results were anonymized prior to analysis and were linked to the facial expression data using nonidentifiable codes. All data were stored securely, with access limited to the research team. Participants received 6000 Japanese yen and reimbursement for transportation costs upon the completion of the experiment. Participants who withdrew from the study before completing the full procedure received compensation proportionate to the duration of their participation in the eligible study procedures.

### Data Analysis

#### Demographics

Continuous variables were compared between the lower cognitive performance group and the HC group using Welch 2-tailed *t* test or the Wilcoxon rank-sum test, as appropriate. Categorical variables were compared between the 2 groups using the Fisher exact test.

#### OpenFace Data Quality

To assess the quality of the OpenFace-derived data, all recordings were visually inspected by a single investigator for apparent recording artifacts that could interfere with facial feature extraction, including facial occlusion, noticeable changes in illumination, head rotation, and the face moving outside the camera field of view. In addition, the frame-level success (1=success, 0=failure) and confidence (range 0‐1) values output by OpenFace were descriptively summarized across all analyzed frames.

#### Video Analysis

##### AU Intensity: Frame-Level Analysis

Because repeated frame-level observations were nested within participants, multilevel models with random intercepts were adopted to account for between-participant variability. In addition, because consecutive video frames are temporally correlated and therefore cannot be regarded as independent observations, an autoregressive correlation structure of order 1 (AR(1)) was incorporated to account for temporal autocorrelation. By accounting for both the clustering of repeated observations within participants and temporal dependence between consecutive frames, this modeling strategy was used to reduce the risk of overestimating statistical evidence due to the large number of correlated frame-level observations.

For each of the 17 AUs, the AU intensity value was used as the response variable, and the group dummy variable (lower cognitive performance group=1, HC=0) was used as the explanatory variable. Because depressive symptoms, age, and sex have been reported to be associated with both facial expressions and cognitive performance [25,33-35], GDS score, age, and sex were included as covariates in all subsequent regression analyses. GDS score and age were standardized using *z*-scores before inclusion in the models.

As already noted, OpenFace AU intensity ranges from 0 to 5. We divided this value by 5 to convert the range to a scale of 0 to 1 so that beta regression analysis can be applied. Beta regression analysis is not applicable when the response variable includes exactly 0 and/or exactly 1. Since the distribution of AU intensity contained unignorable amounts of zeros as shown in the *Results* section, a zero-inflated beta regression model was adopted. This model consists of 2 components. One predicts continuous values strictly between 0 and 1 (beta component), while the other predicts the likelihood of 0 occurring (zero-inflation component). Standardized age, standardized GDS score, and sex were included in both the beta and zero-inflation components. Participant-specific random intercepts and the AR(1) correlation structure were incorporated into both components.

Hereafter, the regression coefficients of the group dummy variable in each component are written as β_beta_ and β_zero_, respectively, using subscripts to identify which component the statistics refer to. Because in both components a logit link is applied to the linear predictor, the fixed effects of the group dummy variable are interpreted as follows: in the beta component, belonging to the lower cognitive performance group is associated with a β_beta_ increase in the logit-transformed mean of AU intensity converted to a value in the range 0 to 1; in the zero-inflation component, belonging to the lower cognitive performance group is associated with a β_zero_ increase in the log-odds of the AU intensity being exactly zero. The analysis was performed using the glmmTMB package [36] in the R statistical software.

Among the 17 AUs, only AU7 (lid tightener) contained observations with intensity values exactly equal to 1. For AU7, the proportion of values exactly equal to 1 was 0.0036 in the lower cognitive performance group and 0 in the HC group, which was substantially lower than the proportion of values exactly equal to 0 as shown in the *Results* section, so for convenience, we subtracted 10^–3^ from the value when it was 1. For AU15 (lip corner depressor), zero intensity values were observed in only 4 participants, all of whom belonged to a single group-by-sex combination (female participants in the lower cognitive performance group). The zero-inflated beta mixed-effects model for AU15 yielded a non-positive-definite Hessian matrix, indicating that reliable parameter estimates could not be obtained. Therefore, for AU15 only, as an alternative analysis, these 4 participants with zero AU15 intensity values were excluded, and a beta mixed-effects regression model was fitted to the remaining participants using the same explanatory variable, covariates, random-effects structure, and AR(1) correlation structure described above.

Because this study was exploratory and hypothesis-generating, aiming to identify candidate AU patterns associated with lower cognitive screening performance, rather than designed to test prespecified confirmatory hypotheses regarding specific AUs, *P* values were not adjusted for multiple comparisons in any of the AU analyses. Unadjusted *P* values are reported for all AU analyses, and the findings should be interpreted as exploratory and require validation in independent samples.

##### AU Intensity: Participant-Level Sensitivity Analysis

To examine whether the findings from the frame-level video analysis of AU intensity were robust to participant-level aggregation and were not driven solely by the large number of correlated frame-level observations, participant-level sensitivity analyses were additionally performed for findings that were significant at the frame level. In these sensitivity analyses, each participant, rather than each individual video frame, constituted the unit of analysis. As in the frame-level video analysis, the group dummy variable was included as the explanatory variable. The proportion of frames with an AU intensity of exactly zero (zero occurrence rate) was calculated for each participant. The resulting participant-level zero occurrence rates were then analyzed using fractional logistic regression with a quasibinomial family and logit link.

##### AU Intensity: Model Diagnostics

Model adequacy was evaluated for the mixed-effects models used in the AU intensity analysis by examining model convergence, optimization warnings, Hessian definiteness, and random effect estimates. Residual diagnostics were performed using the DHARMa package [37], including tests of residual uniformity, dispersion, and zero-inflation.

##### AU Presence: Participant-Level Analysis

We initially attempted to analyze repeated frame-level AU presence values using multilevel logistic regression models with the same explanatory variable, covariates, random-effects structure, and AR(1) correlation structure as those used for the AU intensity analysis. However, these models did not yield reliable parameter estimates for several AUs because of convergence and estimation problems. For this reason, frame-level AU presence values were aggregated within each participant. For each participant, the proportion of frames with AU absence (AU presence =0) was calculated for each AU. For each of the 18 AUs, the zero-occurrence rate was analyzed using fractional logistic regression implemented with a quasibinomial family and logit link. The group dummy variable (lower cognitive performance group=1, HC=0) was used as the explanatory variable, with standardized age, standardized GDS score, and sex included as covariates

### Correspondence Between AU Intensity and AU Presence

In OpenFace, AU intensity and AU presence are generated by separately trained predictors and may not always produce consistent outputs [29], so additional exploratory analyses were performed to examine the correspondence between these 2 measures in the present dataset. Spearman correlation analysis was used to assess the association between AU intensity and AU presence across the analyzed observations. In addition, cross-tabulations were used to examine the correspondence between zero versus nonzero AU intensity values and AU presence (0 vs 1), both across all AUs and for the AU corresponding to the primary finding. Boxplots of AU intensity values stratified by AU presence (0 vs 1) were also examined to further characterize the correspondence between the 2 measures.

### Exploratory Single-Frame Analysis

Even if group differences were observed in video analyses, it would remain unclear whether these differences reflected facial characteristics already present in the initial resting facial appearance or facial movements that became apparent over the course of the recording. To address this issue, an exploratory single-frame analysis was performed using the first analyzed frame from each participant. Because the first 5 seconds of each recording had been excluded to remove transient responses to the experimental instructions, the first analyzed frame was assumed to represent a relatively stable facial appearance.

For the exploratory single-frame analysis, AUs showing group differences in the video analyses were examined, with first-frame AU intensity and AU presence values analyzed separately as response variables, while retaining the same covariates as those used in the video analysis; random effects and autocorrelation were inherently not applicable to the single-frame analysis.

## Results

### Demographics

Data from 5 participants were excluded from the analysis because OpenFace was unable to accurately detect eyebrow positions due to obstructions caused by eyeglasses or bangs. Thus, 31 participants in the lower cognitive performance group and 14 participants in the HC group were included in the analysis. Participants’ demographics are summarized in Table 1, and no significant between-group differences were observed in age, sex, educational level, or GDS score. Detailed results of the group comparisons are provided in Table S1 (Multimedia Appendix 1).

**Table 1.** Participants’ demographics^a^.

Characteristics	Lower cognitive performance (n=31)	HC^b^ (n=14)	*P* value
Age (y), mean (SD)	74.3 (5.7)	73.9 (4.4)	.77
Sex, n			.52
Male	10	6	
Female	21	8	
Education level (y)			.49
≥13	20	11	
<13	11	3	
MoCA-J^c^ total score, mean (SD)	23.7 (1.4)	27.1 (1.3)	―
GDS^d^, median (IQR)	2.0 (1.0-3.5)	1.5 (1.0-3.0)	.82

a Montreal Cognitive Assessment (MoCA) score was not compared between groups because the Montreal Cognitive Assessment was used to define the healthy controls and lower cognitive performance groups. Age was compared using Welch *t* test, and Geriatric Depression Scale score was compared using the Wilcoxon rank-sum test, and sex and education level were compared using the Fisher exact test.

b HC: healthy control.

c MoCA-J: Montreal Cognitive Assessment Japanese version.

d GDS: Geriatric Depression Scale.

### OpenFace Data Quality

Visual inspection identified no apparent recording artifacts that could interfere with facial feature extraction. Throughout the analyzed recordings, the face remained within the camera field of view, and no facial occlusion, noticeable changes in illumination, or head rotation were observed. Across all analyzed video frames from the 45 participants (56,250 frames), the success value was 1 for every frame. The confidence values were 0.98 for 55,827 frames, 0.93 for 422 frames, and 0.88 for 1 frame. Thus, all analyzed frames (56,250 frames) were retained for analyses. These results indicate that AU estimation was performed under technically stable recording conditions.

### Video Analysis

#### AU Intensity: Frame-Level Analysis

The distribution characteristics of AU intensity are presented in Table 2. For the 16 AUs excluding AU15, analyzed using zero-inflated beta mixed-effects models, the fixed effect of the group dummy variable in the beta component was not significant. For the zero-inflation component, the fixed effect of the group dummy variable was significant for the AU10 (upper lip raiser; β_zero_=36.53, SE_zero_=7.41; *P*<.001). For AU15, which was analyzed separately using a beta mixed-effects regression model, the fixed effect of the group dummy variable was also not significant. Additional results are provided in Table S2 (Multimedia Appendix 1).

**Table 2.** Distribution characteristics of action unit (AU) intensity^a^.

Action unit	FACS^b^ name	Lower cognitive performance (n=31)		HC^c^ (n=14)	
		Mean (SD)	Proportion of zeros	Mean (SD)	Proportion of zeros
AU1	Inner brow raiser	0.29 (0.14)	0.008	0.29 (0.16)	0.00006
AU2	Outer brow raiser	0.38 (0.19)	0.080	0.29 (0.19)	0.057
AU4	Brow lowerer	0.09 (0.11)	0.312	0.08 (0.10)	0.366
AU5	Upper lid raiser	0.07 (0.08)	0.322	0.08 (0.10)	0.457
AU6	Cheek raiser	0.19 (0.11)	0.064	0.21 (0.16)	0.046
AU7	Lid tightener	0.45 (0.24)	0.056	0.40 (0.18)	0.002
AU9	Nose wrinkler	0.10 (0.11)	0.290	0.10 (0.10)	0.270
AU10	Upper lip raiser	0.08 (0.10)	0.412	0.14 (0.12)	0.138
AU12	Lip corner puller	0.10 (0.12)	0.355	0.12 (0.11)	0.159
AU14	Dimpler	0.11 (0.12)	0.354	0.11 (0.14)	0.353
AU15	Lip corner depressor	0.26 (0.14)	0.044	0.31 (0.13)	0.000
AU17	Chin raiser	0.05 (0.07)	0.480	0.08 (0.10)	0.240
AU20	Lip stretcher	0.31 (0.16)	0.067	0.29 (0.14)	0.024
AU23	Lip tightener	0.03 (0.04)	0.481	0.04 (0.05)	0.428
AU25	Lips part	0.16 (0.10)	0.071	0.12 (0.14)	0.208
AU26	Jaw drop	0.17 (0.13)	0.145	0.13 (0.14)	0.300
AU45	Blink	0.18 (0.15)	0.169	0.21 (0.12)	0.107

a Action unit intensity values were rescaled from 0-5 to 0-1.

b FACS: Facial Action Coding System.

c HC: healthy control.

#### AU Intensity: Participant-Level Sensitivity Analysis

In the frame-level video analysis of AU intensity, the zero-inflation component showed a significant group difference for AU10. Participant-level zero occurrence rates were therefore analyzed using fractional logistic regression implemented with a quasibinomial family and logit link. A higher zero occurrence rate was observed in the lower cognitive performance group for AU10 (β=1.60, SE =0.77, *t*_40_=2.07; *P*=.045). Thus, the finding observed in the frame-level video analysis for AU10 was retained after participant-level aggregation.

#### AU Intensity: Model Diagnostics

All final AU intensity models converged successfully without optimization warnings and yielded positive-definite Hessian matrices (Table S3 in Multimedia Appendix 1). Detailed DHARMa residual diagnostic results for all AU intensity models are presented in Table S4 (Multimedia Appendix 1).

For the AU10 intensity model corresponding to the primary finding, although the DHARMa uniformity test was statistically significant, no significant deviations were detected in the dispersion or zero-inflation tests. The model also converged successfully without optimization warnings and yielded a positive-definite Hessian matrix. Although a slight deviation was observed in the upper tail of the DHARMa QQ plot for AU10 (Figure S1 in Multimedia Appendix 1), this deviation may partly reflect the limited number of observations in the higher-intensity range. AU10 intensity values were highly concentrated in the lower range of the distribution, with 18,372/56,250 (32.7%) for observations with an AU10 intensity of 0, 4106/56,250 (7.3%) for observations with an AU10 intensity exceeding 0.3, and 1029/56,250 (1.8%) for observations with an AU10 intensity exceeding 0.4.

#### AU Presence: Participant-Level Analysis

The distribution characteristics of AU presence are presented in Table 3. The fixed effect of the group dummy variable was not significant for any of the 18 AUs. Additional model results are provided in Table S5 (Multimedia Appendix 1).

**Table 3.** Distribution characteristics of action unit (AU) presence.

Action unit	FACS^a^ name	Lower cognitive performance (n=31)	HC^b^ (n=14)
		Proportion of zeros	Proportion of zeros
AU1	Inner brow raiser	0.093	0.001
AU2	Outer brow raiser	0.139	0.030
AU4	Brow lowerer	0.917	0.780
AU5	Upper lid raiser	0.693	0.715
AU6	Cheek raiser	0.662	0.523
AU7	Lid tightener	0.418	0.388
AU9	Nose wrinkler	0.999	0.928
AU10	Upper lip raiser	0.829	0.648
AU12	Lip corner puller	0.808	0.819
AU14	Dimpler	0.637	0.701
AU15	Lip corner depressor	0.113	0.066
AU17	Chin raiser	0.906	0.705
AU20	Lip stretcher	0.291	0.094
AU23	Lip tightener	0.647	0.419
AU25	Lips part	0.882	0.905
AU26	Jaw drop	0.780	0.784
AU28	Lip suck	0.925	1.000
AU45	Blink	0.460	0.357

a FACS: Facial Action Coding System.

b HC: healthy control.

### Correspondence Between AU Intensity and AU Presence

Spearman correlation analysis showed a moderate positive correlation between AU intensity and AU presence (Spearman’s *r*=0.59; *P*<.001). To further characterize the correspondence between the 2 measures, cross-tabulations and boxplots were examined (Table S6 and Figure S2 in Multimedia Appendix 1).

Cross-tabulation showed that, across all AU values extracted from the analyzed video frames, 187,204/199,003 (94.07%) for observations with an AU intensity of 0 that were classified as AU presence=0, 366,010/757,247 (48.33%) for observations with nonzero AU intensity values that were classified as AU presence=0, and 11,799/199,003 (5.93%) for observations with an AU intensity of 0 that were classified as AU presence=1, indicating that disagreement between the 2 measures was primarily attributable to observations with nonzero AU intensity values being classified as AU presence =0.

Similar patterns were observed for the primary finding, AU10. Among observations with nonzero AU intensity values, 25,117/37,878 (66.31%) for AU10 observations with nonzero intensity that were classified as AU presence=0, and 23/18,372 (0.13%) for AU10 observations with zero intensity that were classified as AU presence=1. The boxplots further illustrated that observations classified as AU presence=0 generally had lower AU intensity values than those classified as AU presence =1.

Together, these findings indicate that although AU intensity and AU presence were moderately correlated, subtle facial movements reflected in low but nonzero AU intensity estimates may not always be captured as present by the binary AU presence output. This classification discrepancy may partly contribute to the different findings for AU10 between the AU intensity and AU presence analyses.

### Exploratory Single-Frame Analysis

Because the primary video analysis finding for AU10 intensity reflected a group difference in the probability of zero values, additional single-frame analyses were conducted for AU10 by dichotomizing the first-frame intensity values as zero versus nonzero, with zero coded as 1 and nonzero as 0. These values were analyzed using logistic regression. The corresponding first-frame AU presence values were also analyzed using logistic regression. No significant group differences were observed for AU10 in the logistic regression analyses of either the dichotomized first-frame intensity values (β=1.93, SE=1.17, *z*=1.65; *P*=.10) or the corresponding first-frame AU presence values (β=−1.22, SE =0.85, *z*=−1.44; *P*=.15).

## Discussion

### Principal Findings

The present study compared AUs during fixation-cross viewing between older adults classified as having lower cognitive screening performance based on the MoCA and HCs. The principal finding was that older adults with lower cognitive screening performance exhibited a higher zero occurrence rate for AU10 (upper lip raiser) in the AU intensity analysis. Given that low-intensity AU activations were frequently classified as absent by the binary AU presence output, AU intensity may capture facial information that is not fully reflected by AU presence alone. The higher zero occurrence rate for AU10 in the lower cognitive performance group was consistently observed in both the frame- and participant-level analyses. This consistency suggests that the observed group difference was not driven solely by the large number of correlated frame-level observations. Furthermore, the exploratory single-frame analysis showed no significant group differences for AU10, suggesting that the observed differences may reflect facial movements occurring during fixation-cross viewing, rather than static facial appearance alone.

### Comparisons to Prior Work

Similar to the findings of Ko et al [25], a wide range of AUs was detected during fixation-cross viewing under neutral facial conditions, and most AU intensity values were concentrated in the lower range of the distribution (Table 2). This consistency suggests that the detection of AU intensity across a wide range of AUs under neutral facial conditions is not unique to the present study.

Previous studies [15-17,19,20] investigating facial characteristics associated with cognitive impairment have primarily analyzed facial expressions obtained during conversations or emotion-eliciting tasks, when facial behaviors may be influenced by social interaction and emotional processes. In contrast, our findings suggest that facial characteristics associated with lower cognitive screening performance are not limited to conversational or emotion-eliciting contexts but can also be observed during fixation-cross viewing, where the influence of social interaction and explicit emotional stimulation is minimized. Furthermore, Kameyama et al [21] reported that AI can distinguish individuals with dementia from HCs using static photographs of neutral facial expressions. However, because these approaches relied on black-box algorithms, it has remained unclear which specific facial regions or characteristics contributed to these classifications and whether these characteristics reflected stable facial appearance characteristics, such as wrinkles, or facial movements reflected in the images. By analyzing facial videos using interpretable AU-based measures, the present study provides additional insight into this question by showing that the facial characteristic associated with cognitive screening performance involved AU10, which reflects movement in the mouth region, and may reflect spontaneous facial movement around the mouth during fixation-cross viewing rather than static facial appearance.

### Implications

One possible explanation for the reduced spontaneous movements around the mouth suggested by the AU10 finding is related to changes in social engagement. For example, lower cognitive screening performance may be associated with fewer opportunities for social engagement [38]. This reduction may result in less frequent use of facial movements around the mouth during daily communication, which may, in turn, contribute to the subtle difference in perioral facial movements suggested by the present findings. Alternatively, reduced social engagement itself may be associated with both lower cognitive screening performance and reduced facial movements around the mouth, although the direction of these associations remains unclear. Because the present study did not investigate the mechanisms underlying these associations, future studies incorporating measures of social engagement and other potential contributing factors are warranted to clarify the mechanisms underlying the observed facial characteristics.

The present findings identify candidate facial biomarkers associated with lower cognitive screening performance. These candidate facial biomarkers may provide a scientific foundation for future digital prescreening approaches intended to encourage older adults with possible but unrecognized cognitive decline to undergo comprehensive cognitive assessment. Because the fixation-cross viewing task requires only simple instructions and does not involve conversation or emotion-eliciting stimuli, it may provide a practical paradigm for future studies investigating facial-information–based digital prescreening. Collectively, these findings contribute to a better understanding of the facial characteristics associated with cognitive decline and may facilitate future investigations of interpretable facial biomarkers.

### Limitations

The present study has several limitations. First, participants were classified according to MoCA-J scores, rather than a comprehensive clinical diagnosis. Therefore, the identified facial characteristics should be interpreted as candidate facial biomarkers associated with lower cognitive screening performance, rather than biomarkers specific to clinically diagnosed MCI. Future studies should examine whether these findings can be generalized to populations with clinically diagnosed MCI.

Second, because the present study aimed to identify candidate facial biomarkers rather than to develop or validate a classification model, diagnostic metrics such as sensitivity, specificity, accuracy, area under the receiver operating characteristic curve, and external validation were beyond the scope of this study, and screening performance was not evaluated.

Third, several potential confounding factors were not systematically assessed, including medical conditions and medication use other than those specified in the exclusion criteria, head pose and camera-to-face distance, and physiological or subjective stress during fixation-cross viewing. In addition, because the primary finding involved a mouth-related AU, the potential influence of oral and dental conditions should also be considered. Future studies should systematically evaluate the influence of these factors.

Fourth, automated facial expression analysis in older adults has limitations. Older faces remain underrepresented in datasets used to train facial expression analysis algorithms, which may affect estimation performance [39]. In addition, OpenFace-derived AU estimates should not be regarded as direct measures of facial muscle activity. Future studies combining OpenFace with facial electromyography in older adults are needed.

Fifth, the relatively small and imbalanced sample may have limited statistical power and increased the influence of individual variability. Although the AU10 finding was consistent across the frame-level and participant-level analyses, independent replication in larger samples is needed.

Sixth, because multiple AUs were examined without adjustment for multiple comparisons, the possibility of false-positive findings should be considered. Accordingly, the statistical findings should be interpreted as exploratory and hypothesis-generating.

Finally, although facial expressions show substantial cross-cultural commonality, culturally related differences have also been reported [40]. Because all participants were Japanese, replication in independent samples from different cultural backgrounds is needed. In addition, self-selection bias should be considered because individuals who were reluctant to provide facial images may have chosen not to participate.

### Conclusions

The present study suggests that 1 facial characteristic associated with lower cognitive screening performance was observed as an interpretable AU during a simple fixation-cross viewing condition without conversation or emotion-eliciting tasks. In particular, the observed facial characteristic involved AU10 (upper lip raiser), reflecting differences in facial changes around the mouth. These findings suggest candidate facial biomarkers that may provide a scientific foundation for future research on facial information-based digital prescreening.

## Supplementary material

10.2196/99948 Multimedia Appendix 1 Participant characteristics, facial action unit intensity and presence analyses, and model diagnostics.

## Abbreviations

AD: Alzheimer disease
AU: action unit
EEG: electroencephalography
FACS: Facial Action Coding System
GDS: Geriatric Depression Scale
HC: healthy control
MCI: mild cognitive impairment
MoCA-J: the Japanese version of the Montreal Cognitive Assessment
MRI: magnetic resonance imaging
